# Assessing Ebola-related web search behaviour: insights and implications from an analytical study of Google Trends-based query volumes

**DOI:** 10.1186/s40249-015-0090-9

**Published:** 2015-12-10

**Authors:** Cristiano Alicino, Nicola Luigi Bragazzi, Valeria Faccio, Daniela Amicizia, Donatella Panatto, Roberto Gasparini, Giancarlo Icardi, Andrea Orsi

**Affiliations:** Department of Health Sciences (DISSAL), University of Genoa, Genoa, 16132 Italy; Hygiene Unit, IRCCS AOU San Martino – IST of Genoa, Genoa, 16132 Italy

**Keywords:** Ebola, Google Trends, Human Development Index, Internet, Relative search volume, Web, West Africa

## Abstract

**Background:**

The 2014 Ebola epidemic in West Africa has attracted public interest worldwide, leading to millions of Ebola-related Internet searches being performed during the period of the epidemic. This study aimed to evaluate and interpret Google search queries for terms related to the Ebola outbreak both at the global level and in all countries where primary cases of Ebola occurred. The study also endeavoured to look at the correlation between the number of overall and weekly web searches and the number of overall and weekly new cases of Ebola.

**Methods:**

Google Trends (GT) was used to explore Internet activity related to Ebola. The study period was from 29 December 2013 to 14 June 2015. Pearson’s correlation was performed to correlate Ebola-related relative search volumes (RSVs) with the number of weekly and overall Ebola cases. Multivariate regression was performed using Ebola-related RSV as a dependent variable, and the overall number of Ebola cases and the Human Development Index were used as predictor variables.

**Results:**

The greatest RSV was registered in the three West African countries mainly affected by the Ebola epidemic. The queries varied in the different countries. Both quantitative and qualitative differences between the affected African countries and other Western countries with primary cases were noted, in relation to the different flux volumes and different time courses. In the affected African countries, web query search volumes were mostly concentrated in the capital areas. However, in Western countries, web queries were uniformly distributed over the national territory. In terms of the three countries mainly affected by the Ebola epidemic, the correlation between the number of new weekly cases of Ebola and the weekly GT index varied from weak to moderate. The correlation between the number of Ebola cases registered in all countries during the study period and the GT index was very high.

**Conclusion:**

Google Trends showed a coarse-grained nature, strongly correlating with global epidemiological data, but was weaker at country level, as it was prone to distortions induced by unbalanced media coverage and the digital divide. Global and local health agencies could usefully exploit GT data to identify disease-related information needs and plan proper communication strategies, particularly in the case of health-threatening events.

**Electronic supplementary material:**

The online version of this article (doi:10.1186/s40249-015-0090-9) contains supplementary material, which is available to authorized users.

## Multilingual abstracts

Please see Additional file 1 for translations of the abstract into the six official working languages of the United Nations.

## Background

The 2014 Ebola epidemic in West Africa, primarily affecting Guinea, Sierra Leone and Liberia, was the largest Ebola outbreak since the first epidemic in Sudan and Zaire in 1976. Since December 2013, Ebola has infected almost 28,000 people, mostly in West Africa, resulting in more than 11,400 deaths and attracting public interest worldwide [1]. This led to millions of Ebola-related Internet searches being performed during the period of the epidemic, making Ebola the most searched item on Yahoo engine and the fifth most searched term on Google in 2014 [2, 3]. Indeed, Internet represents a quickly growing source of health data with millions of people seeking healthcare information on the web daily [4].

Internet search data have been recently exploited both for public health surveillance purposes and for analysing the public’s searching behaviour as a reaction to infectious disease outbreaks [5]. In particular, trend data generated by the number of Google searches – the most popular search engine worldwide – can be investigated by Google Trends (GT), a freely accessible tool that provides data on geospatial and temporal patterns in search volumes for user-specified terms [6]. Google Trends offers valuable insights into population behaviour and health-related phenomena, especially in the area of infectious diseases, as well as in the areas of mental health and substance use, non-communicable diseases and other general behaviours [6].

A systematic review published in 2014 by Nuti et al. highlighted that in recent years researchers have been increasingly utilising GT for a diversity of health topics with some successful applications in the field of infectious disease surveillance, especially in countries with high Internet penetration levels [7]. For instance, GT has been efficaciously used to develop a model, known as Google Flu Trends, which is able to provide early warning of increases in influenza-like illness (ILI) incidence one to two weeks before traditional influenza and ILI surveillance systems, but with some limitations [8, 9].

Recently, Jena et al. found a strong correlation between searches for human immunodeficiency virus (HIV) and the United States of America (US) Centers for Disease Control and prevention (CDCs) HIV incidence rates, and were able to construct a model based on web searches for the period 2007–2008 to accurately predict HIV incidence for 2009–2010 [10]. Other authors demonstrated that GT could be used to forecast the peak of scarlet fever in the United Kingdom (UK) five weeks before its arrival [11].

Google Trends has also proven to be valuable for assessing tropical diseases, such as malaria, in countries with low levels of Internet penetration [12]. Moreover, Google search queries have been demonstrated to be effective and accurate at predicting, at the national-level, periods of high incidence of dengue in countries with underdeveloped surveillance systems, such as Bolivia, Brazil, India, Indonesia, Singapore and Mexico [13–15].

Furthermore, analysing Internet query volumes for specific terms using GT allows for the quantification and interpretation of interest levels in health topics at the population level [7]. For example, Cha and Stow exploited GT to assess the social relevance, collective knowledge and public perception of an environmental accident, showing that the event raised public attention on associated environmental issues [16]. Further, search engine query data from Google have been used to assess the public interest in influenza in the year of pandemic and the following year in the UK, Lyme disease in the US and Mycoplasma pneumoniae infection in Finland [17–19].

With respect to the current Ebola epidemic, Househ analysed public interest in Ebola. For this purpose, the author used GT Ebola-related query volumes from the period between 30 September and 30 October 2014, evidencing two peaks of Google searches: the first related to a *TIME* magazine report about the Hazmat crew that boarded a plane after a man said he may be infected with the deadly Ebola virus; the second was associated with a press release from President Obama calling on the National Guard reserves to help to contain the Ebola virus [20].

However, no prior studies have investigated Google search queries related to the 2014 Ebola outbreak since the very beginning of the epidemic, and for its entire period. Therefore, the current study aimed to evaluate and interpret Google search queries for terms related to the 2014 Ebola outbreak both at the global level and in all countries where primary cases of Ebola occurred. The study also endeavoured to look at the correlation between the number of overall and weekly web searches and the number of overall and weekly new cases of Ebola.

## Methods

Google Trends, an online tracking system of Internet hit-search volumes that recently merged with its sister project Google Insights for Search (Google Inc.), was used to explore Internet activity related to the Ebola epidemic. The portal determines the proportion of searches for user-specified terms among all searches performed using Google. It then provides a relative search volume (RSV), which is the query share of a particular term for a given location and time period, normalised by the highest query share of that term over the time series and presented on a scale from 0 to 100. Each point of the graph generated by GT is divided by the highest point, which is conventionally set at 100.

Google Trends was mined from 29 December 2013 to 14 June 2015 to cover the entire period of the Ebola outbreak. “Ebola” and “Ebola”-related terms were searched also in local languages of the three most affected West Africa countries. For each keyword, the queries were performed with a keyword being used both as the “search term” and the “search topic”. Using the first option, GT searched the exact string of text typed by the user, while using the second option all searches related to the query and automatically suggested by GT were included; this latter option is still under testing (beta function). It is possible to search for up to five queries each time [21].

We descriptively analysed the changes in web search queries at the global level and in all countries where primary cases of Ebola were registered (namely, Guinea, Sierra Leone, Liberia, Nigeria, Mali, Senegal, US, Spain, UK and Italy) over the entire period of the outbreak, and evaluated the correlation with news headlines reported using Google News. Furthermore, we analysed top queries and rising queries related to the search term or search topic, and assessed both the interest at global level and the regional interest at country level in Ebola on the web.

Pearson’s correlation was performed to correlate Ebola-related RSVs with the number of Ebola cases retrieved from the World Health Organization (WHO) database, available at http://apps.who.int/gho/data/node.ebola-sitrep (last accessed on 17 August 2015) [1]. Numbers of weekly and overall cases of Ebola were used for the correlation. In particular, both data from the WHO patient databases and situation reports were analysed. The strength of correlation was measured using the system described by Taylor [22].

Multivariate regression was performed using Ebola-related RSV as a dependent variable, and epidemiological data (overall number of Ebola cases) and the Human Development Index (HDI) as predictor variables. This led to the creation of different mathematical models. The HDI was used to correct raw GT data, enabling a comparison to be made among the different countries and socio-economic contexts.

The HDI was retrieved from the official website of the United Nations Development Programme, available at http://hdr.undp.org/en/2014-report (last accessed on 17 August 2015). The HDI is a composite statistical indicator, which combines life expectancy, education and income indices as proxies of country development. The Index is not based on economic growth alone and is able to capture the potential development of a country. The HDI was preferred to the Internet Penetration Index because the latter underweights the real use of the Internet, especially in low- and middle-income countries, where access to the web is limited and occurs mainly from public places such as Internet points and cyber cafes [23]. Moreover, in recent years, the use of mobile phones has increased dramatically, particularly in Sub-Saharan Africa, igniting a revolution in Internet access [24]. It has been demonstrated that a strong positive correlation exists between the number of mobile phone subscribers and HDI, especially in African countries [25]. It is also noteworthy that a positive correlation exists between HDI and Internet usage or penetration in Africa, although it is not as significant as it is in Europe and other developed countries [26]. The last available data for HDI (year 2013) were used.

Computation was done using SPSS software v21.0.0 (IBM Corporation, Armonk, NY, US). Values ≤0.05 were considered statistically significant. All data on the number of Ebola cases and the GT index are available as supplementary material.

## Results

**Fig. 1:**
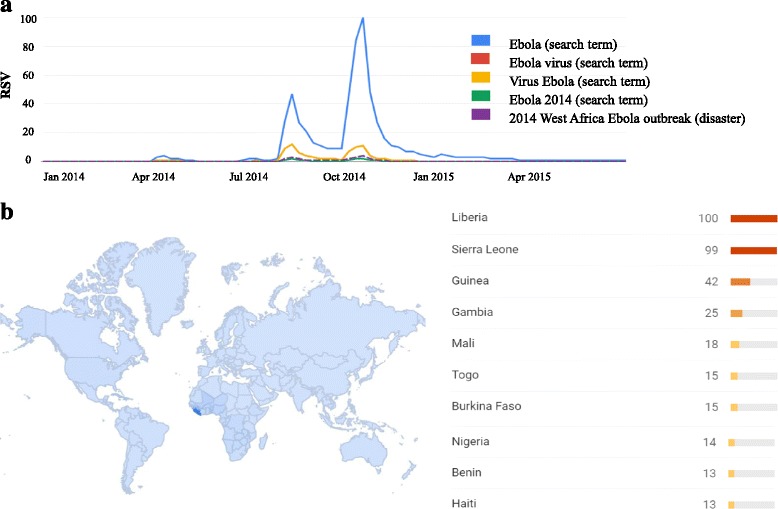
**a** Google Trends curve as RSVs for “Ebola” and Ebola-related terms (as “search term” or “search interest”) from December 2013 to June 2015; **b** Regional interest heat map for Ebola-related activities worldwide

Figure 1a shows the first five queries generating the highest RSV values at a global level, namely “Ebola”, “Ebola virus”, “Virus Ebola”, “Ebola 2014” and “2014 West Africa Ebola outbreak”. The keyword that yielded the greatest RSV was “Ebola”, searched using the “search term” option. “Ebola” was searched seven times more than the second-listed query (data not shown). Considering the term “Ebola”, the date of 16 October 2014 had the highest index score of 100: on this particular day, President Obama issued a press release calling upon the National Guard reserves to help to contain the Ebola virus. A smaller peak (RSV = 46/100), registered on 8 August 2014, corresponded with the headline in the *Belfast Telegraph* “WHO declares Ebola emergency”. Despite quantitative differences in terms of RSV, the curves generated by the five listed queries observed a similar trend over time. Other searches conducted using the “search topic” option, or in local languages using the “search term” option, did not yield any detectable RSV (data not shown).

With respect to regional interest in the search term “Ebola” (see Fig. 1b), the greatest RSV was registered in Liberia (100/100), followed by Sierra Leone (99/100) and Guinea (42/100), the three West African countries mainly affected by the 2014 Ebola epidemic. In relation to other countries where Ebola primary cases were registered, the RSVs were 18/100, 14/100, 11/100, 5/100, 4/100, 3/100 and 2/100 in Mali, Nigeria, Senegal, US, Spain, UK and Italy, respectively.

Table 1 shows the top and rising queries for the search term “Ebola” both at the global and country levels. Queries varied in the different countries and with respect to global researches: in particular, three out of the first five global Ebola-related rising queries were related to the first primary case of Ebola in the US.

**Table 1:** Ebola-related search queries at the global and national levels, December 2013 – June 2015

Country	Top Ebola-related queries	RSV	Ebola-related rising queries	Increase (%)
Global	virus ebola	100	dallas ebola	breakout
	ebola symptoms	30	ebola 2014	breakout
	ebola news	20	ebola texas	+850 %
	el ebola	20	ebola patient	+400 %
	ebola outbreak	20	ebola in us	+300 %
Liberia	liberia ebola	100	ebola in liberia	breakout
	ebola in liberia	65	ebola news	breakout
	ebola virus	45	ebola outbreak	breakout
	ebola news	40	ebola outbreak liberia	breakout
	liberia news ebola	25	ebola update	breakout
Sierra Leone	ebola sierra leone	100	about ebola	breakout
	ebola update	50	ebola cases	breakout
	ebola news	35	ebola latest news	breakout
	ebola virus	20	ebola new	breakout
	about ebola	20	ebola news	breakout
Guinea	virus ebola	100	ebola conakry	breakout
	ebola guinee	95	ebola en guinee	breakout
	ebola en guinee	55	ebola en guinée	breakout
	ebola guinée	55	ebola guinee	breakout
	fievre ebola	50	ebola guinée	breakout
Mali	ebola mali	100	ebola au mali	breakout
	virus ebola	50	ebola mali	breakout
	ebola au mali	45	fievre ebola	breakout
	fievre ebola	20	virus ebola	breakout
Nigeria	ebola virus	100	about ebola	breakout
	ebola nigeria	50	ebola cure	breakout
	ebola in nigeria	40	ebola disease	breakout
	what ebola	25	ebola in nigeria	breakout
	ebola news	20	ebola latest	breakout
Senegal	ebola senegal	100	ebola au senegal	breakout
	ebola virus	85	ebola dakar	breakout
	ebola au senegal	30	ebola senegal	breakout
	ebola dakar	25	ebola virus	breakout
	guinee ebola	20	guinee ebola	breakout
United States	ebola virus	100	ebola 2014	breakout
	ebola symptoms	50	ebola dallas	breakout
	ebola outbreak	40	ebola nurse	breakout
	what is ebola	35	ebola texas	+750 %
	ebola us	30	ebola usa	+450 %
Spain	el ebola	100	caso ebola	breakout
	ebola españa	75	ebola 2014	breakout
	ebola en españa	50	ebola en españa	breakout
	noticias ebola	35	ebola enfermera	breakout
	ebola virus	30	ebola españa	breakout
Italy	virus	100	caso ebola	breakout
	ebola virus	95	ebola 2014	breakout
	ebola italia	95	ebola africa	breakout
	ebola sintomi	80	ebola in italia	breakout
	ebola in italia	40	ebola italia	breakout
United Kingdom	virus ebola	100	ebola in uk	breakout
	ebola symptoms	65	ebola nurse	breakout
	ebola uk	65	ebola uk	+400 %
	ebola news	40	ebola news	+250 %
	what is ebola	40	bbc news ebola	+180 %

**Fig. 2:**
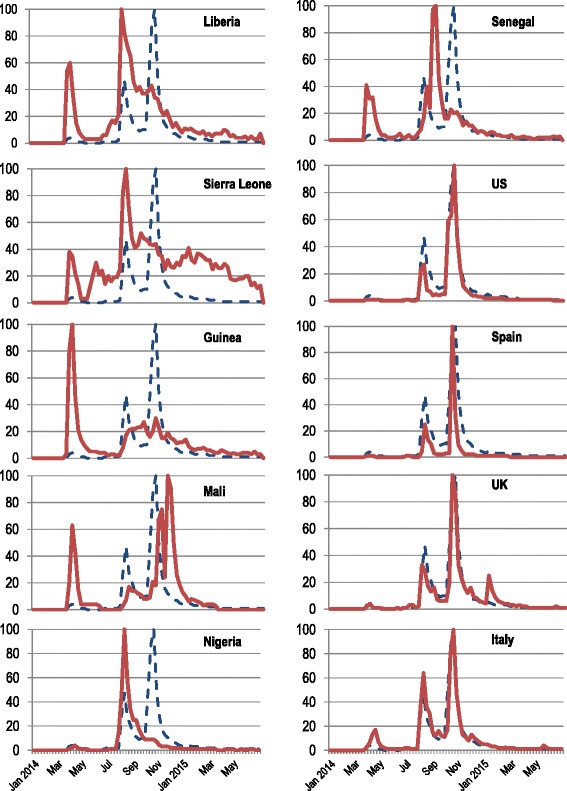
Google Trends curve as RSV for “Ebola” from December 2013 to June 2015 searched worldwide (dotted blue line), and in all countries where primary cases of Ebola were registered (red lines)

Figure 2 shows the interest in Ebola, using “Ebola” as a search term, over time both at the global and country levels. Both quantitative and qualitative differences between the affected African countries and other Western countries with primary cases were noted, in terms of the different flux volumes (expressed as relative percentages) and different time courses.

In the African countries, the peaks coincided with Ebola victims being quarantined in Guinea and the WHO announcement of the Ebola epidemic. In particular, in Liberia, Sierra Leone, Nigeria and Senegal, the highest peak of web searches (RSV = 100/100) corresponded with the *Belfast Telegraph* headline “WHO declares Ebola emergency”; in Guinea the highest peak coincided with the news of the first Ebola victims quarantined; and in Mali with the news of an American doctor being cured for Ebola. In the three primarily affected African countries, Ebola-related web searches declined in the months after the WHO announcement of the Ebola epidemic, despite the increase in Ebola cases.

In all Western countries, a similar pattern in Ebola-related web searches was observed. Further, these trends reflected the global interest in Ebola: the two highest peaks of web searches (on 16 October 2014 and 8 August 2014, corresponding to US President Obama’s press release and the WHO announcement of the Ebola epidemic, respectively) could be detected in all curves. In the UK and Italy, two further peaks were observed: in UK, a small peak (RSV = 25/100) was registered on 30 December 2014, corresponding to the news headline “Ebola patient in Britain transferred to London”; while in Italy a small peak (RSV = 17/100) was registered on the third week of April 2014, some days after the news of the first Ebola victims quarantined in Guinea.

**Fig. 3:**
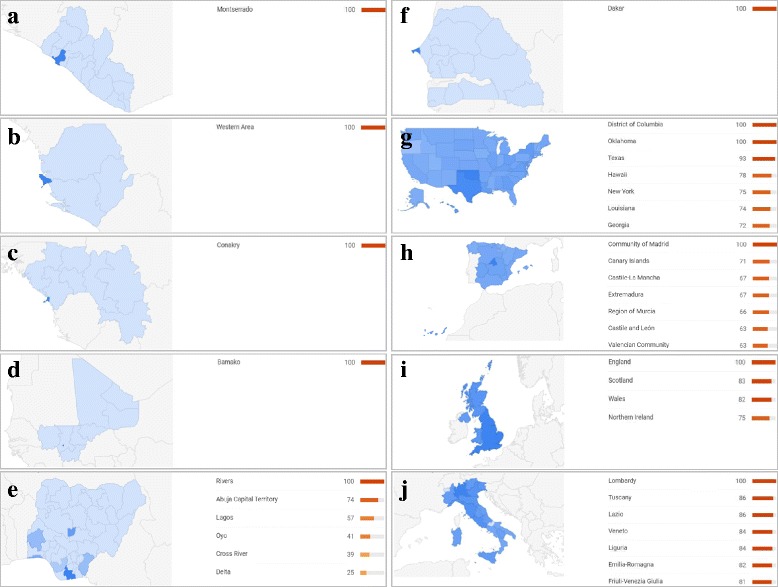
Regional interest heat map for Ebola-related activities in all countries where primary cases of Ebola were registered over the entire period of the outbreak; **a**) Guinea, **b**) Sierra Leone, **c**) Liberia, **d**) Mali, **e**) Nigeria, **f**) Senegal, **g**) US, **h**) Spain, **i**) UK, **j**) Italy

Figure 3 outlines the regional interest in the search term “Ebola” in each of the 10 countries affected by Ebola primary cases. In the affected African countries, with the exception of Nigeria (see Fig. 3e), web query search volumes were concentrated in the capital areas, while no queries were reported in regions when the epidemic first spread. In Nigeria, the interest in Ebola was concentrated not only in the capital (Abuja), but also in other not neighbouring regions. However, in Western countries, the web queries were uniformly distributed over the national territory (see Fig. 3).

**Fig. 4:**
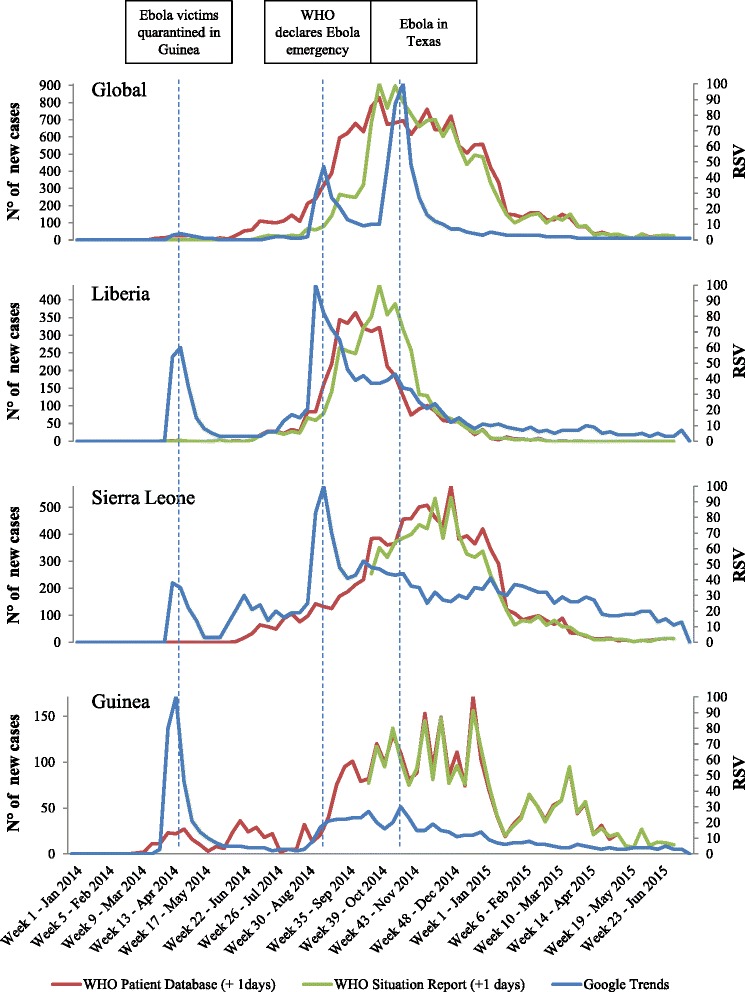
Correlation between GT curves as weekly RSVs for “Ebola” from December 2013 to June 2015, searched worldwide (upper), Liberia, Sierra Leone and Guinea (below), and the weekly number of new Ebola cases

Figure 4 shows the weekly GT index for search queries for “Ebola” compared with the number of new weekly Ebola cases, as reported in the WHO patient database and situation report, at the global level and in the three mainly affected West African countries. Raw data about weekly GT index for search queries for "Ebola" and new weekly Ebola cases, as reported in the WHO patient database and situation report, are available in Additional file 2. The correlation between the number of global new weekly cases of Ebola and the weekly GT index was moderate, both considering the WHO patient database (*r* = 0.553, *p*-value <0.001) and the WHO situation report (*r* = 0.409, *p*-value = 0.001). In terms of the three countries mainly affected by the Ebola epidemic, the correlation between new weekly cases of Ebola reported in the WHO patient database and the weekly GT index varied from weak in Guinea (*r* = 0.232, *p*-value = 0.07) to moderate in Sierra Leone (*r* = 0.481, *p*-value <0.001) and Liberia (*r* = 0.640, *p*-value <0.001). When correlating with new weekly cases of Ebola reported in the WHO situation report, the correlation was weaker and not statistically significant in all cases.

**Fig. 5:**
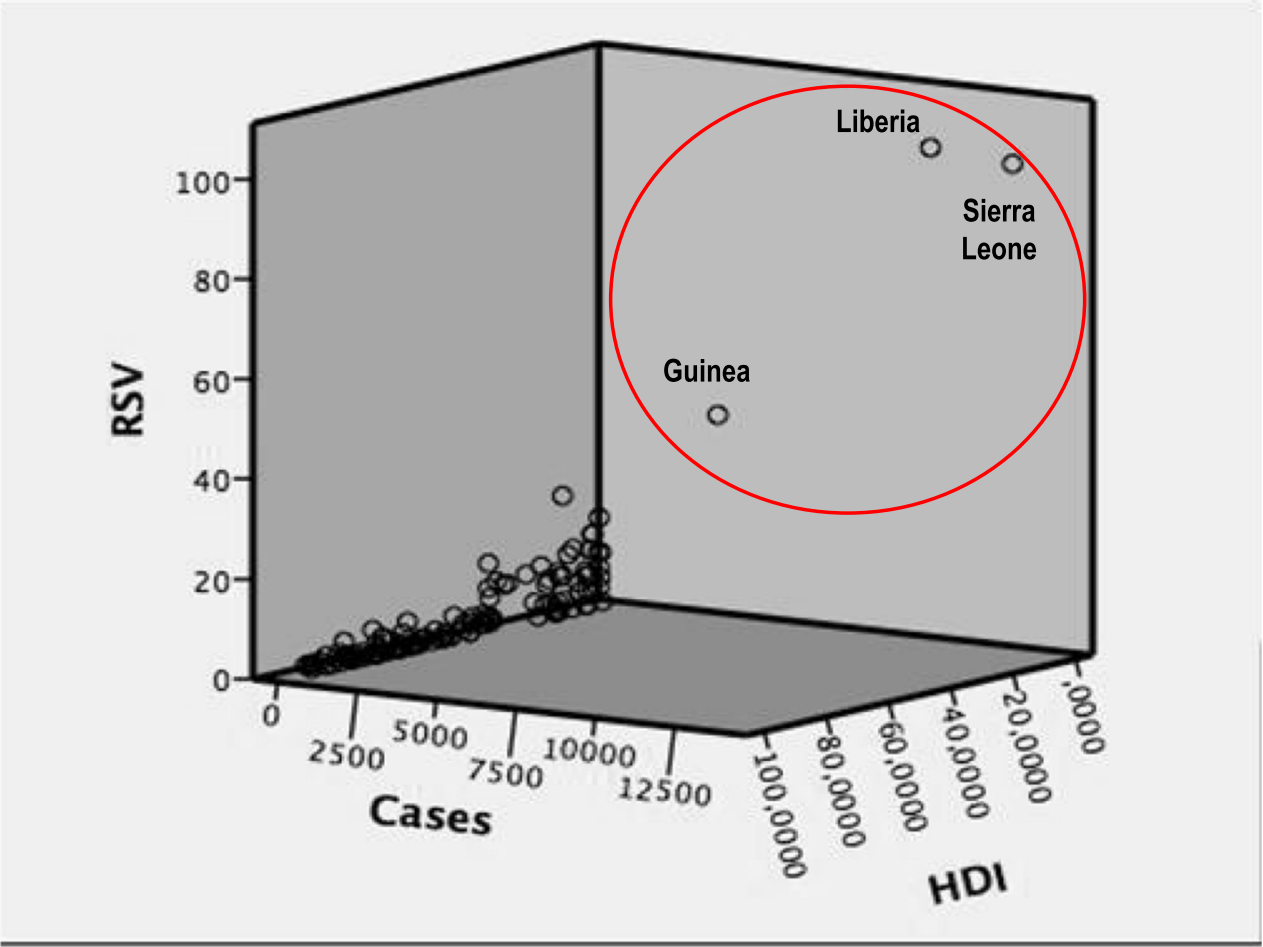
Scatterplot visualising the correlation between RSV for “Ebola” from December 2013 to June 2015, the total number of Ebola cases per country registered over the entire period of the outbreak, and the HDI in all countries where primary cases of Ebola were registered

Figure 5 shows the correlation between the RSV for “Ebola”, the total number of Ebola cases per country registered over the entire period of the outbreak and the HDI, in all countries where Ebola cases were registered.

The correlation between the number of Ebola cases registered in all countries during the study period and the GT index was very high (*r* = 0.916; *p*-value <0.001). In particular, the three mainly affected African countries are characterised by a strong correlation between the RSV, the number of Ebola cases and the HDI, which is quite different from the other countries (see Fig. 5).

In order to investigate these correlations more deeply, two different regression models were developed, using GT-based RSVs for “Ebola” searched worldwide, and epidemiological data (the number of Ebola cases) (first model), and the number of Ebola cases and the HDI (second model) as predictor variables (see Table 2). The second model showed the best fit (Akaike information criterion = 709.927). The HDI was a negative predictor for GT-based RSV (*p*-value <0.0001), whilst the number of Ebola cases (*p*-value <0.0001) and the interaction between the number of cases and the HDI (*p*-value <0.0001) were positive predictors.

**Table 2:** Regression models using GT-based RSVs for “Ebola” searched worldwide, and epidemiological data (the number of Ebola cases) and the HDI as predictor variables

Parameter	Estimate	Std error	t	Sig.	95 % CI	
					Lower limit	Upper limit
FIRST MODEL AIC 776.979						
Intercept	4.083293	.381482	10.704	.000	3.328576	4.838010
Ebola cases	.008047	.000255	31.552	.000	.007542	.008551
SECOND MODEL AIC 709.927						
Intercept	7.771741	.596617	13.026	.000	6.590580	8.952901
Ebola cases	.006199	.000466	13.301	.000	.005277	.007122
HDI	-.078454	.010805	-7.261	.000	-.099846	-.057061
Ebola cases X HD	.000543	.000147	3.693	.000	.000252	.000834I

## Discussion

This study is the first analysis of web search behaviour related to the 2014 Ebola outbreak, both in quantitative and qualitative terms, for the entire epidemic period. Previous published studies have investigated GT and Twitter activities related to the Ebola epidemic, but only over a restricted time period [20, 27, 28].

The Ebola epidemic has attracted global public interest, generating millions of related Internet searches worldwide. The peak of global Internet search related to the term “Ebola” coincided with news of the first US Ebola case at the end of September 2014; by contrast the news about the first victims quarantined in Guinea, which appeared in March 2014 in the *San Francisco Gate* newspaper, as indicated by GT and Google News, did not globally generate a valuable number of web searches. Searches for “Ebola” performed in October 2014 were 76 times higher than those performed in March 2014, when reports of the Ebola outbreak first appeared. According to GT, “the early reports in March caused a surge, but it was when cases started to appear outside of Africa that searches hit a global scale. People around the world searched to know more as news gathered momentum” [3]. Notably, in the three West African countries mainly affected by the epidemic, Ebola-related activities declined after there were spikes in web searches associated with the news of the first victims quarantined in Guinea and the WHO declaration of the Ebola emergency, despite the increase in Ebola cases.

Our findings suggest that web search interest in Ebola (including in the African countries), both at the global and national levels, was strongly influenced by traditional, electronic and social media coverage in the Western countries.

As already discussed by other authors, media coverage during the 2014 Ebola outbreak was unbalanced and occasional [29]. For instance, as argued by an editorial in the *Lancet*, in the US disproportionate airtime was given to the nine confirmed American cases of Ebola compared with the massive human crisis occurring in the three West African countries mainly affected by the epidemic [29]. Data about worldwide traffic on Twitter and Google about Ebola, collected in the period between September and November 2014, demonstrated a dramatic increase in web searches, as news spread about the first US case diagnosed on 30 September 2014 [30]. Prior to this, daily tweets containing the word “Ebola” were, on average, very low [27]. More recently, a cross-section survey of Twitter data and GT data, conducted from 30 September to 29 October 2014, confirmed that Twitter and electronic news outlets focused more on the possible threat of Ebola reaching the US than on Ebola awareness and educational campaigns [20]. Towers et al., by using the “news media contagion model” and fitting Twitter and GT data, demonstrated that the massive media coverage absolutely disproportionate to the real threat to public health in the US contributed to generating web search activities and tweets [31].

These observations have important implications for global public health. For instance, the public concern – as communicated by traditional and innovative media – that Ebola could reach Western countries may have contributed to expedite the research for an Ebola vaccine. By contrast, even though there were already hundreds of Ebola cases in West Africa during the first months of 2014 and the disease was responsible, in the same continent, for numerous outbreaks in the last three decades, until this threat “hit the headlines” in developed countries, it represented a neglected disease both for the general public and public health practitioners and researchers [32].

Furthermore, the media coverage in developed countries seemed to substantially influence not only the temporal trend of web searches but also the topics of the search queries at the global level. Three out of the first five global Ebola-related rising queries are related to the first primary case Ebola in the US, reflecting the global public interest in the possible threat of Ebola reaching the US rather than in the real epidemic taking place. Even in this case, search topics about Ebola seem to be influenced by media coverage in Western countries.

However, when considering the topic queries at the national level, Ebola-related searches appeared to be “country specific”: Google users who responded to Ebola-related news by searching Google and the relative volume of the search topics in different countries could reflect Ebola-related news itself. Interestingly, during the epidemic, very few queries were related to educational information about the disease, or to environmental and personal hygiene measures to prevent the transmission of Ebola (see Table 1). We can speculate that this could have contributed to the failure in adopting proper preventive measures and favoured considerable misunderstanding about the risks of exposure to Ebola.

From an epidemiological standpoint, the analysis of correlation between the number of Ebola cases and GT-based RSV demonstrated that using web search volume related to the single term “Ebola” for surveillance purposes may not be feasible. Regression analyses performed at a global level enabled us to separate the three mainly affected West African countries from the other countries in terms of web search behaviour (see Table 2 and Fig. 5). Further, our correlation results showed that Ebola-related RSV per country matched with the number of cases registered in that given country for the entire epidemic period, even though the degree of accuracy varied depending on (a) the country and (b) the considered timeline. When considering the number of weekly cases of Ebola, the correlation between RSVs and epidemiological data was weaker.

In fact, as the relative volume of Internet searches is likely to be correlated with the perceived information needs of Internet users, web searches may be useful for predicting epidemic outbreaks. This may be particularly pertinent if the majority of relevant internet searches are made as a result of information seeking, based on the users’ own or friends’ symptoms, instead of simply searching for something novel that is read in the daily news.

To date, GT has been successfully used as an integrative tool of a traditional surveillance system for epidemics and infectious diseases, with endemic diffusion at the country level, exhibiting seasonal patterns, and receiving relatively constant and not distortive media attention. Moreover, it is currently better suited to track disease activity in developed countries, as it requires large populations of web search users to be most effective [7, 33]. However, countries with relatively low Internet penetration can also accumulate sufficient search queries to build surveillance models based on GT, as demonstrated by Chan and colleagues [13].

In particular, GT served as a base for the development of predicting models for the surveillance of influenza and dengue. These models were designed using an automated method that selected – among millions of the most common Google web search queries – a combination of ILI and dengue-related searches (i.e. queries about symptoms, complications, remedies, etc.) that could most accurately predict the percentage of visits to CDCs associated with ILIs in each US region, and official WHO dengue case count data used for each of the analysed countries [8, 13]. A similar approach has effectively estimated malaria activity in Thailand in the period 2005–2010 [14].

Interestingly, the predicting model for dengue detected and removed “spurious spikes” in web search time series, as they could not reflect the real spread of the disease, but could be determined by “panic-induced searching” when media coverage about a particular outbreak acts as an amplifier of web-search activity [13].

Therefore, for a panic-inducing event such as an Ebola outbreak, media reaction strongly influenced the volume of Google queries, especially in Western countries, generating “spurious spikes” in web search time series that prevent the use of web disease-related single term queries for epidemiological surveillance purposes. A similar situation occurred during the outbreak of avian influenza, which spread from China to Turkey between 2005 and 2006: although no outbreaks of avian influenza were reported in the US, the spikes observed for worldwide and US search volume index for the term “bird flu” were superimposable, probably as a reaction to US media reports of an outbreak in Asia [33].

The unfeasibility of GT as an epidemiological predictor could also be explained, at least partially, by the digital divide, as well as by the impact of media coverage [34, 35]. The use of digital technologies to make queries about health is not particularly widespread in the African continent, despite differences among countries, between rural and urban areas, and the rapid evolution of this whole issue. The usage of new technologies in African countries is challenged and sometimes jeopardised by political, economic, social and historical issues, such as ethnic conflicts and wars, and the lack of an adequate infrastructure [36]. Guinea, Sierra Leone and Liberia are indeed characterised by very low Internet penetration levels and the total of the queries are concentrated in the capital areas. Among the African countries, Nigeria is a notable exception. We could speculate that the contrasting web search-related behaviour in this country could have contributed, at least partially, to containing Ebola [28].

Notably, GT, together with social media, could be usefully exploited by global and local health agencies, as well as by public health practitioners, as a tool to identify disease-related information needs, social and behavioural barriers to infection control, and misinformation, as well as to better understand sentiment and risk perception associated with health-threatening events such as an Ebola outbreak [5, 37]. Indeed, analyses of the temporal trend of GT-based query volumes together with their geographic distribution and main topics of searches can furnish a quantifiable and valuable measure of public attention and information needs about a particular communicable disease.

The Internet represents a great opportunity for public health agencies to disseminate healthcare-related information quickly, effectively and cheaply, provided that reliable news are shared with as much of the population as possible, both in developed and developing countries, where the diffusion of new technologies among the population is rapidly increasing. Google Trends – a publicly available data source – could be used as a proxy of the proper diffusion of strategies based on health education messages, allowing to fill the translation gap between best evidence (what some experts know) and practice (what most people do or believe) [38]. Moreover, it could be exploited to determine the level of uptake of information about a public health event. Even during the 2014 Ebola outbreak, some stakeholders leveraged new technologies to plan proper communication strategies and address public concern [39, 40].

Our study had some limitations. Google Trends only captures the search behaviour of a certain segment of the population – those with Internet access and those using Google rather than other search engines (although Google is the most common search engine). However, the major limitation of GT is the lack of detailed information on the method by which Google generates this search data and the algorithms it employs to analyse it. Google Trends data were available only in the form of relative volume and not as absolute values, enabling further data handling and manipulation. Furthermore, temporal changes in the interface and capabilities of GT over time are not documented, which may lead to variations in the search output and in turn the study findings. Thus, the interpretation of the findings may be affected depending on the time and geographical area of the GT data.

Finally, although some privacy issues exist in using Google Web search data, tracing individuals that conduct online searches when signed into their accounts and recording and analysing data about users’ characteristics, such as gender and age, intent of web search and “search outcomes” could improve the usefulness of this tool for public health and health education purposes.

## Conclusion

This is the first study to investigate the online global and local response during the entire period of the 2014 Ebola epidemic, in terms of web search behaviour related to term “Ebola”.

In this study, GT showed a coarse-grained nature, strongly correlating with global epidemiological data, but was weaker at country level, as it was prone to distortions induced by unbalanced media coverage and the digital divide.

Global and local health agencies could usefully exploit GT data to identify disease-related information needs and plan proper communication strategies, particularly in the case of such health-threatening events.

## Additional files

Additional file 1:
**Multilingual abstracts in the six official working languages of the United Nations.** (PDF 346 kb) Additional file 2: S1. Dataset of raw weekly GT data and weekly new cases of Ebola as according to the WHO patient database and situation report at the global level (Sheet 1); in the three main affected West African Ebola countries (Guinea – Sheet 2, Sierra Leone – Sheet 3 and Liberia – Sheet 4); raw weekly GT data in all countries where primary cases of Ebola were registered and Pearson’s correlations (Sheet 5); total GT RSV and the total number of Ebola cases per country (Sheet 6). (XLSX 44 kb)

## Abbreviations

CDC: Center for Disease Control and Prevention
GT: Google Trends
HDI: Human Development Index
HIV: Human immunodeficiency virus
ILI: Influenza-like illness
RSV: Relative search volume
UK: United Kingdom
US: United States of America
WHO: World Health Organization.
