# Supplementary material for: Assessing Ebola-related web search behaviour: insights and implications from an analytical study of Google Trends-based query volumes
Source: Infect Dis Poverty. 2015 Dec 10;4:54. doi: 10.1186/s40249-015-0090-9 (PMC4674955; doi:10.1186/s40249-015-0090-9)
Supplement: Additional file 1: — Multilingual abstracts in the six official working languages of the United Nations. (PDF 346 kb) [file 40249_2015_90_MOESM1_ESM.pdf]

Translation of the abstract into the six official working languages of the United Nations

تقييم السلوك البحثي عن وباء الإيبولا على الإنترنت: الرؤى و الآثار حسب الدراسة التحليلية لأحجام الاستعلام استنادا إلى تقنية الـ Google Trends

Cristiano Alicino, Nicola Luigi Bragazzi, Valeria Faccio, Daniela Amicizia, Donatella Panatto, Roberto Gasparini, Giancarlo Icardi, Andrea Orsi

الملخص

الخلفية: استقطب وباء الإيبولا سنة 2014 اهتمام الجماهير في جميع أرجاء العالم، و هو ما أدّى إلى ملايين الأبحاث على الأنترنت حول الإيبولا في الوقت الذي كان فيه الوباء منشرا. كان الهدف من هذه الدراسة تقييم و شرح استعلامات البحث على موقع Google عن الألفاظ التي لها علاقة بتفشّي الإيبولا على مستوى العالم و في جميع البلدان التي ظهرت فيها حالات الإيبولا الأولى. كما سعت الدراسة أيضا إلى إلقاء نظرة على العلاقة بين العدد الكلّي و الأسبوعي للأبحاث على الإنترنت و العدد الكلّي و الأسبوعي للحالات الجديدة من الإيبولا.

الطرق: تمّ استخدام تقنية الـ Google Trends (GT) لدراسة نشاط الإنترنت المتعلّق بالإيبولا. امتدّت فترة الدراسة من 29 كانون الأوّل/ ديسمبر 2013 إلى 14 حزيران/ يونيو 2015. تمّ تطبيق معامل ارتباط بيرسون للرّبط بين أحجام البحث النسبية (RSVs) المتعلّقة بالإيبولا و عدد حالات الإيبولا الأسبوعية و الكلّيّة. كما تمّ تطبيق الارتداد متعدد المتغيّرات باستخدام أحجام البحث النسبية كمتغيّر تابع، و تمّ استخدام العدد الكلّي لحالات الإيبولا و مؤشّر التنمية البشرية كمتغيّرات تنبؤية.

النتائج: تمّ تسجيل أحجام البحث النسبية الأكبر في ثلاثة بلدان تقع في غرب إفريقيا تفشى فيها داء الإيبولا. كانت الاستعلامات متغيّرة في مختلف البلدان. تمت ملاحظة فروقات كميّة و نوعية بين البلدان الإفريقية التي تفشى فيها الوباء و البلدان الغربية الأخرى التي ظهرت فيها حالات أوّلية، و ذلك يرجع إلى أحجام التدفق المختلفة إلى جانب الدورات الزمنية المختلفة. في البلدان الإفريقية التي تفشّى فيها الوباء، كانت أحجام البحث الاستعلامية على الإنترنت مركّزة في العواصم. لكن في البلدان الغربية، كانت الأبحاث الاستعلامية موزعة بشكل موحد عبر التراب الوطني. بالنسبة لأكثر ثلاثة بلدان تفشى فيها وباء الإيبولا، اختلف الارتباط بين عدد حالات الإيبولا المسجّلة و مؤشّر الـ GT من ضعيف إلى معتدل. كان الارتباط بين عدد حالات الإيبولا المسجّلة في جميع البلدان أثناء فترة الدراسة و مؤشّر الـ GT عاليا جدّا.

الخلاصات: أظهرت تقنية الـ Google Trends طبيعة خشنة، مرتبطة بشكل كبير بالبيانات الوبائية العالمية، لكنها كانت أضعف على مستوى البلدان، حيث أنها كانت عرضة للاختلالات التي تسببت فيها التغطية غير المتوازنة للإعلام و الفجوة الرقمية. كان بإمكان الهيئات الصحية العالمية و المحليّة استغلال بيانات الـ GT بشكل مفيد لتحديد الاحتياجات إلى المعلومات المتعلقة بالوباء و التخطيط لاستراتيجيات تواصل ملائمة، خاصة عند وجود أحداث تهدّد الصحّة.

Translated from English version into Arabic by Mokhtar Oussama Alliouche, through

TRANSLATORS
WITHOUT BORDERS

埃博拉相关的网络搜索行为评估：基于谷歌趋势搜索量分析研究的机制与意义

Cristiano Alicino, Nicola Luigi Bragazzi, Valeria Faccio, Daniela Amicizia, Donatella Panatto, Roberto Gasparini, Giancarlo Icardi, Andrea Orsi

摘要

引言：2014 年西非埃博拉暴发引发了全球公众关注，导致在暴发期间数百万埃博拉相关的网络搜索。本研究的目的在于评估和解释在全球层面和疫情发生国家与埃博拉相关的谷歌搜索问题，并分析总搜索量及每周搜索量与总病例数及每周发病数之间的关系。
方法：采用谷歌趋势（GT）研究 2013 年 12 月 29 日至 2015 年 6 月 14 日与埃博拉相关的互联网活动。采用皮尔森相关性（Pearson’s correlation）分析埃博拉相关搜索量（RSVs）与埃博拉周病例数及总病例数之间的关系。以埃博拉相关的 RSV 为因变量、总病例数和人类发展指数为自变量开展多变量回归分析。
结果：在受埃博拉暴发影响最严重的 3 个西非国家的 RSV 最大。在不同国家间的搜索量差异较大。受暴发影响的非洲国家与存在原发病例的其他西方国家之间存在定量和定性的差异，这种差异与不同搜索量和不同时间段有关。在受暴发影响的非洲国家，网络搜索量大多集中在首都地区。然而，在西方国家，网络搜索均匀地分布于整个国家范围。在受埃博拉暴发影响最严重的 3 个非洲国家，每周新发埃博拉病例数与每周 GT 指数之间的相关性由弱变为中等。在研究期间所有国家报告埃博拉病例数与 GT 指数之间高度关联。
结论：谷歌趋势显示了一个粗糙的特性，它与全球流行病学数据高度相关，但在国家级层面的相关性较低，因媒体覆盖率和数字鸿沟 GT 易失真。全球和地方卫生机构可有效地利用 GT 数据，以确定疾病相关信息需求，制定合适的沟通策略，特别是在出现威胁公众健康的情况下。

Translated from English version into Chinese by Chen Tian-mu, edited by Yang Pin, through

CHINESE CENTER FOR DISEASE CONTROL AND PREVENTION
NATIONAL INSTITUTE OF PARASITIC DISEASES

Evaluation du comportement des utilisateurs du web dans les recherches d’informations sur Ebola: Découvertes et répercussions d’après une étude analytique des grandes tendances sur Google.

Cristiano Alicino, Nicola Luigi Bragazzi, Valeria Faccio, Daniela Amicizia, Donatella Panatto, Roberto Gasparini, Giancarlo Icardi, Andrea Orsi

Résumé

Contexte: l’épidémie Ebola de 2014 en Afrique de l’Ouest a attiré l’attention du public dans le monde entier, donnant lieu, sur Internet, à des millions de recherches liées à Ebola, effectuées pendant la période de l’épidémie. L’objectif de l’étude a été d’évaluer et d’interpréter les recherches d’informations sur Google pour des termes afférents à l’épidémie Ebola, aussi bien au niveau mondial que dans tous les pays où s’étaient produits les premiers cas d’Ebola. L’étude s’est également employée à observer la corrélation existant entre le nombre de recherches Internet globales et hebdomadaires, et le nombre de nouveaux cas globaux et hebdomadaires d’Ebola.
Méthodes: Google Trends (GT) a été utilisé pour explorer l’activité Internet afférente à Ebola. La période de l’étude va du 29 Décembre 2013 au 14 Juin 2015. La corrélation de Pearson a été interprétée pour mettre en corrélation le volume de recherches relatives à Ebola (VRR) avec le nombre de cas hebdomadaires et globaux d’Ebola. La régression multivariée a été interprétée en utilisant le VRR comme une variable dépendante, et le nombre global de cas d’Ebola et l’Indice du Développement Humain ont été utilisés comme variables prédictives.
Résultats: le plus grand VRR a été enregistré dans les trois pays d’Afrique de l’Ouest principalement touchés par l’épidémie Ebola. Les demandes d’informations ont varié dans les différents pays. Des différences tant quantitatives que qualitatives entre les pays africains et les autres pays occidentaux ayant eu des premiers cas ont

été observées, concernant le volume de flux et les différentes périodes de temps. Dans les pays africains touchés, le volume des demandes d'informations a été principalement concentré dans les régions de la capitale. Cependant, dans les pays occidentaux, les demandes d'informations sur le web ont été uniformément réparties sur le territoire national. S'agissant des trois pays principalement touchés par l'épidémie Ebola, la corrélation entre le nombre de nouveaux cas hebdomadaires et l'indice GT hebdomadaire a été variable, allant de faible à modérée. La corrélation entre le nombre de cas d'Ebola enregistrés dans tous les pays au cours de la période de l'étude et l'indice GT a été très élevée.

Conclusions: Google Trends a montré une nature à gros grains, en forte corrélation avec les données épidémiologiques mondiales, mais a été plus faible au niveau du pays, car il a été sujet à des distorsions induites par une couverture médiatique mal équilibrée et le fossé du numérique. Les organismes de santé mondiaux et locaux ont pu exploiter utilement les données de GT pour identifier les besoins d'informations afférents à la maladie, et planifier des stratégies adéquates de communication, en particulier dans le cas d'événements menaçants pour la santé.

Translated from English version into French by Ode Laforge, through

TRANSLATORS
WITHOUT BORDERS

Оценка поведения при связанном с Эбола поиске в Интернет: Оценки и выводы из аналитического исследования объемов запросов на основе Google Trends

Кристиано Аличино, Никола Луиджи Брагацци, Валериа Фаччио, Даниэла Амичициа, Донателла Панатто, Роберто Гаспарини, Джанкарло Икарди, Андреа Орси

Аннотация

Предисловие: Эпидемия Эбола в Западной Африке 2014-го года вызвала общественный интерес во всем мире, что привело к миллионам поисков в Интернет, связанных с Эбола, в период эпидемии. Целью настоящего исследования является оценка и интерпретация запросов на поиск в Google по терминам, связанным со вспышкой Эбола, как на глобальном уровне, так и во всех тех странах, где произошли первичные случаи Эбола. В исследовании сделана попытка рассмотрения корреляции между числом общих и недельных поисков в Интернет и числом общих и недельных новых случаев Эбола.

Методы: Для исследования связанной с Эбола Интернет-активности использовалась Google Trends (GT). Периодом исследования было время от 29 декабря 2013 г. до 14 июня 2015 г. Для выяснения корреляции относительных объемов поисков (RSV), связанных с Эбола, с числом недельных и общих случаев Эбола выполнялась корреляция Пирсона. Выполнялась многомерная регрессия с использованием связанной с Эбола RSV как зависимой переменной, а в качестве предсказываемых переменных использовались общее число случаев Эбола и индекс человеческого развития.

Результаты: Наибольший RSV был зарегистрирован в трех странах Западной Африки, главным образом, пострадавших от эпидемии Эбола. Запросы варьировали по разным странам. Были отмечены как

количественные, так и качественные различия между пострадавшими африканскими странами и другими западными странами с первичными случаями в отношении различных объемов потока и различных периодов течения. В пострадавших африканских странах объемы поисковых запросов в Интернет концентрировались в основном в столичных регионах. В западных странах, однако, запросы в Интернет были равномерно распределены по территории страны. Что касается трех наиболее пострадавших от эпидемии Эбола стран, то корреляция между числом новых недельных случаев Эбола и недельным индексом GT варьировала от слабой до умеренной. Корреляция между числом случаев Эбола, зарегистрированных во всех странах в период исследования, и индексом GT была очень высокой.
Выводы: Google Trends выявила крупнозернистый характер, сильно коррелированный с глобальными эпидемиологическими данными, но более слабый на страновом уровне, поскольку тот был подвержен искажениям, вносимым несбалансированным освещением в медиа и «цифровым неравенством». Глобальные и местные агентства здравоохранения могут успешно использовать данные GT для выявления связанных с заболеванием информационных потребностей и планирования надлежащих стратегий коммуникации, особенно в случае угрожающих здоровью событий.

Translated from English version into Russian by Alexander Somin, through

TRANSLATORS
WITHOUT BORDERS

Análisis del comportamiento de búsqueda sobre el ébola en la web: Percepciones e implicaciones de un estudio analítico de los volúmenes de consultas basados en tendencias en Google

Cristiano Alicino, Nicola Luigi Bragazzi, Valeria Faccio, Daniela Amicizia, Donatella Panatto, Roberto Gasparini, Giancarlo Icardi, Andrea Orsi

Resumen

Antecedentes: La epidemia de ébola del año 2014 en África Occidental atrajo el interés del público a nivel mundial., Esto resultó en millones de búsquedas relacionadas con ébola en Internet que se llevaron a cabo durante el período de la epidemia. El objetivo de este estudio fue evaluar e interpretar las consultas de búsqueda en Google para términos asociados con el brote de ébola tanto a nivel mundial como en todos los países donde ocurrieron los casos primarios de ébola. El estudio también intentó observar la correlación entre la cantidad de búsquedas totales y por semana en la web y la cantidad de nuevos casos de ébola totales y por semana.
Métodos: Se utilizó Google Trends (GT) para explorar la actividad en Internet asociada al ébola. El período de estudio fue del 29 de diciembre de 2013 al 14 de junio de 2015. Se utilizó el coeficiente de correlación de Pearson para relacionar los volúmenes de búsquedas relativas (VBR) asociadas al ébola con la cantidad de casos totales y por semana de ébola. Se realizó regresión multivariante mediante el uso de una variable dependiente de VBR asociadas al ébola, y luego se utilizó la cantidad total de casos de ébola y el Índice de Desarrollo Humano como variables predictoras.

Resultados: La mayor VBR se registró en los tres países de África Occidental principalmente afectados por la epidemia del ébola. Las consultas variaron en los distintos países. Se notaron diferencias cuantitativas y cualitativas entre los países africanos afectados y otros países occidentales con casos primarios, con relación a los distintos volúmenes de flujo y distintos transcursos de tiempo. En los países de África afectados, los volúmenes de consultas de búsqueda en la web se concentraron principalmente en las zonas de las ciudades capitales. Sin embargo, en los países occidentales, las consultas en la web se distribuyeron uniformemente en todo el territorio nacional. En términos de los tres países principalmente afectados por la epidemia del ébola, la correlación entre la cantidad de nuevos casos de ébola por semana y el índice de GT semanal, varió de débil a moderada. La correlación entre la cantidad de casos de ébola registrados en todos los países durante el período de estudio y el índice de GT fue muy alta.

Conclusiones: Google Trends mostró información de naturaleza aproximada, que se correlaciona significativamente con la información epidemiológica a nivel mundial, pero que fue más débil a nivel de país, ya que estaba sujeta a distorsiones inducidas por una cobertura desequilibrada de los medios y la brecha digital. Las agencias de salud mundiales y locales podrían explotar la información de GT de manera útil para identificar las necesidades de información asociada a enfermedades y planificar estrategias de comunicación adecuadas, en especial en el caso de eventos de atentan contra la salud.

Translated from English version into Spanish by Maria Alejandra Aguada, through

TRANSLATORS
WITHOUT BORDERS
